# Restoration of Glymphatic Influx After Photothrombotic Stroke Using Low‐Intensity Focused Ultrasound

**DOI:** 10.1111/cns.70451

**Published:** 2025-06-02

**Authors:** Ming‐Yen Hsiao, Yu‐Ling Lin, Meng‐Ting Lin, Wei‐Hao Liao, Wen‐Shiang Chen, Chueh‐Hung Wu

**Affiliations:** ^1^ Department of Physical Medicine and Rehabilitation, College of Medicine National Taiwan University Taipei Taiwan; ^2^ Department of Physical Medicine and Rehabilitation National Taiwan University Hospital Taipei Taiwan; ^3^ Departments of Physical Medicine and Rehabilitation National Taiwan University Hospital Hsin‐Chu Branch Hsinchu Taiwan

**Keywords:** cerebrospinal fluid, glymphatic, low‐intensity focused ultrasound, stroke

## Abstract

**Aims:**

This study aimed to investigate the regional modulation effect of low‐intensity focused ultrasound (LIFU) on cerebrospinal fluid (CSF) influx dynamics, focusing on the differential changes between hemispheres in a mouse model of unilateral ischemic stroke.

**Methods:**

LIFU was administered with a commercial transducer at the unilateral M1 cortex of mice using specific parameters (0.5 MHz center frequency, 250 kHz pulse repetition frequency, 300 ms burst duration, 2 ms pulse length, 50% intraburst duty factor, 2 s interstimulation interval, spatial peak temporal average intensities of 0.5 W/cm^2^, and a total sonication duration of 10 min). The regional CSF influx was observed through in vivo macroscopy and brain section imaging in a photothrombotic stroke model, both with and without LIFU.

**Results:**

Localized LIFU stimulation at the M1 region triggered a rapid CSF influx, peaking 20–30 min poststimulation. Following photothrombotic stroke, a marked reduction in CSF influx was observed in both ipsilesional and contralesional MCA regions, most notably on Days 1 and 3, with a return to baseline by Day 7. However, daily contralesional LIFU stimulation for 7 days effectively restored CSF influx on both sides.

**Conclusion:**

Daily contralesional LIFU stimulation restores impaired CSF influx bilaterally after stroke, suggesting potential therapeutic implications for stroke‐related pathophysiology.

## Graphical abstract

We examine immediate effect and 7‐day LIFU on CSF influx in stroke mice. LIFU rapidly increased CSF influx, peaking at 20–30 min post‐stimulation. Stroke reduced CSF influx, lowest at day 1‐3, with recovery by day 7. Daily contralesional LIFU restored CSF influx, suggesting stroke recovery benefits.

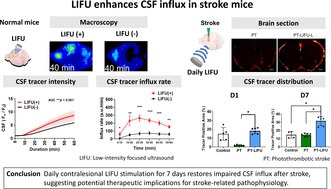

## 1 Introduction

Stroke is one of the leading causes of mortality and morbidity, causing neurological deficits such as hemiplegia, aphasia, dysphagia, and reducing quality of life [1]. The glymphatic system is a circulating network constituting the influx of cerebrospinal fluid (CSF) along periarterial spaces into the brain parenchyma and interstitial fluid back into the perivenous space, a vital mechanism for clearing metabolic waste and maintaining fluid homeostasis [2]. The dysfunction of this system has been associated with various central nervous system (CNS) pathologies including stroke, traumatic brain injury, hydrocephalus, and Alzheimer's disease [2, 3, 4, 5, 6]. In a mouse model of middle cerebral artery occlusion (MCAO), impaired CSF influx into the injured cortex was observed, but it was reversible following recanalization, indicating that the alteration of the glymphatic system is one of the key features in stroke [4]. Another study of MCAO revealed that CSF influx occurred along the enlarged perivascular space, with an accelerated rate of influx during the first few minutes of stroke onset that is related to brain edema [5]. In a human MRI study, the diffusion tensor imaging along perivascular spaces (DTI‐ALPS) index, reflecting the glymphatic function, was significantly lower at the ipsilesional hemisphere within 14 days after ischemic stroke [6]. Although the exact changes in the glymphatic system after stroke remain unclear, these findings suggest its dysfunction is closely linked to ischemic stroke pathology.

Transcranial focused ultrasound (tFUS), with the advantages of high spatial resolution and deep targeting, has emerged as a new‐generation brain stimulation tool [3]. Based on the intensity, high‐intensity tFUS exerts thermal ablation for oncological treatment [7], and further developed an FDA‐approval device for essential tremor and Parkinson's disease [8]. Meanwhile, low‐intensity focused ultrasound (LIFU) with microbubbles, operating at a similar range to diagnostic ultrasound levels (< 1–1.5 MPa), has also been developed for blood–brain barrier (BBB) opening with numerous undergoing clinical trials [3]. Additionally, LIFU without microbubbles may have neuromodulatory effects, directly activating neurons or modulating cortical excitability, as demonstrated in animal and human studies using behavioral, neurophysiological, and functional imaging assessments [9, 10]. Studies in healthy individuals revealed that tFUS stimulation to the primary motor cortex (M1) decreased cortical excitability, as indicated by reduced motor‐evoked potential (MEP) amplitudes and attenuated or enhanced intracortical facilitation [11, 12]. Apart from modulating cortical excitability, transcranial LIFU has been shown in murine models to enhance the CSF influx from the perivascular space into the brain parenchyma, potentially promoting glymphatic circulation [13, 14]. Recent studies have shown that tFUS combined with microbubbles enhances the movement of fluorescent tracer through the perivascular space and into the interstitial space [15, 16], likely as a result of ultrasound‐induced vessel deformation [15].

In disease models, LIFU has demonstrated positive effects. In a mouse model of Alzheimer's disease, LIFU enhances the clearance of beta‐amyloid waste via the glymphatic system [17]. In an MCAO rat model, LIFU could reduce the infarct area observed in MRI scans and decrease brain damage as seen in histopathological analyses, indicating a neuroprotective effect [18]. Similarly, in an MCAO mouse model, LIFU stimulation resulted in improved neurobehavioral outcomes, reduced brain infarct volume, and modulation of microglia polarization [19]. Additionally, it was reported to mitigate BBB disruption and brain edema formation [20].

Currently there are no available studies that investigate the modulation effect of LIFU on the glymphatic system after ischemic stroke. Therefore, in this study, we aimed to investigate the efficacy of LIFU on CSF influx dynamics in a stroke mouse model by in vivo real‐time imaging. We hypothesize that glymphatic circulation is impaired after ischemic stroke, manifesting as decreased CSF influx, and can be partially restored after LIFU stimulation.

## 2 Methods and Methods

### 2.1 Animals

All animal experimental procedures were conducted in accordance with the care and use guidelines of the Laboratory Animal Center at National Taiwan University College of Medicine and were approved by the Institutional Animal Care and Use Committee (IACUC, approval No. 20201142) of National Taiwan University College of Medicine. Male C57BL/6 mice (body weight 20–25 g) purchased from NARLabs were used in this study. Animals were randomized using a computer‐based random order generator.

### 2.2 LIFU Stimulation

Transcranial LIFU was applied through a commercial focused ultrasound transducer (H‐104, Sonic Concepts, USA) placed at the M1 cortex of the brain (1 mm posterior and 3.5 mm lateral to the bregma) using the following parameters: center frequency = 0.5 MHz, pulse repetition frequency (PRF) = 250 kHz, pulse train duration = 300 ms, pulse duration = 2 ms, duty cycle of pulse train = 50%, interstimulation interval = 2 s, and spatial peak temporal average intensities (I_spta_) = 0.5 W/cm^2^, total pulse train repetition duration = 10 min. These parameters were chosen based on previous LIFU neuromodulation studies [21].

### 2.3 CSF Tracer Distribution in Brain Section

Thirty minutes post intracisternal infusion, mice were euthanized, and brains were fixed with 4% cold paraformaldehyde in PBS overnight. 500 μm coronal slices were cut using a vibratome (MicroSlicer DTK‐1000 N, DSK, Kyoto, Japan) and incubated in 2% phosphate‐buffered saline with Tween (PBST) for 2 days to enhance tissue permeability. After three PBS washes, tissues were cleared with RapiClear 1.49 (SunJin Lab Co., Hsinchu City, Taiwan) for 1 h, promoting transparency. Samples were imaged using an Olympus BX51 fluorescence microscope with ToupTek camera and extended depth of field (EDF) method (ToupView software, Hangzhou, China) for improved image quality and faster information acquisition. Tracer influx (area %) was analyzed using ImageJ software (16‐bit image type; analyze particle setting: 80–infinity μm^2^ size range, 0.0–1.0 circularity, and automatic threshold). Paravascular influxes were counted in brain sections where subarachnoid tracer extended into the parenchyma, and influx length was measured from the subarachnoid space to the distal end using ToupView software.

### 2.4 In Vivo Macroscopic Imaging

In each experiment, the CSF tracer (BSA‐Alexa Fluor 647, A34785, Invitrogen) was injected into the mouse's cisterna magna after LIFU treatment. The cisterna magna, located between the skull base and spinal cord, was identified by dissecting the hind neck muscle, and the needle was inserted using a stereotaxic procedure. Subsequently, 5 μL of the tracking agent BSA‐Alexa Fluor 647 (5 mg/mL) was injected at a rate of 1 μL per minute via an electric micro‐syringe.

For in vivo transcranial macroscopic imaging (MVX10, Olympus, Tokyo), the entry of CSF tracers into the brain (influx) was observed. Imaging was conducted at 30‐s intervals over 0 to 60 min posttracer injection, utilizing an ORCA‐spark digital CMOS camera (C11440‐36 U, Hamamatsu, Japan) and cellSens Standard 3 software (Olympus, Japan). Circular regions of interest (ROIs) with a radius of approximately 1.604 mm (area = 8.021 mm^2^) were drawn for analysis.

### 2.5 Photothrombotic Stroke Model

The mice are consistently anesthetized with 1.5% isoflurane, receive subcutaneous Carprofen (5 mg/kg) for pain relief, and are intraperitoneally injected with rose bengal (100 mg/kg). A scalp incision is made, and the M1 area (1 mm posterior and 3.5 mm lateral to the bregma) is exposed to 561 nm laser irradiation at 50 mW for 15 min. The laser is positioned at a height of 5 cm from the mice's skull to induce local thrombosis.

### 2.6 CSF Influx After Photothrombotic Stroke

The change of CSF influx was observed in a mouse model after inducing photothrombotic stroke. The CSF influx was examined by macroscopy 10 min after the induction of photothrombotic stroke, with or without LIFU stimulation. Images were recorded at 30‐s intervals for 60–90 min when the fluorescence intensity reached maximal plateau. Due to the overt scalp incision and the presence of behavioral stroke activity, it was not possible to blind the experimenter to the animal's treatment group.

### 2.7 Statistical Analysis

All numerical data are expressed as mean ± standard error of the mean (SEM) and analyzed by GraphPad Prism software (Prism 8.0.2, GraphPad Software, La Jolla, CA, USA). The Shapiro–Wilk test was used to test for normality. Statistical significance was assessed using the independent *t*‐test or its nonparametric equivalent, the Mann–Whitney *U* test, for comparisons between two groups. Due to non‐normality in the data, the Kruskal–Wallis test was employed to assess overall differences among groups, followed by post hoc Dunn test. For all studies, a *p* value < 0.05 was considered statistically significant. The statistical analyses and sample sizes used for each experiment were specified in the figure legends.

## 3 Results

### 3.1 LIFU Enhances CSF Tracer Distribution and Penetration

**FIGURE 1:**
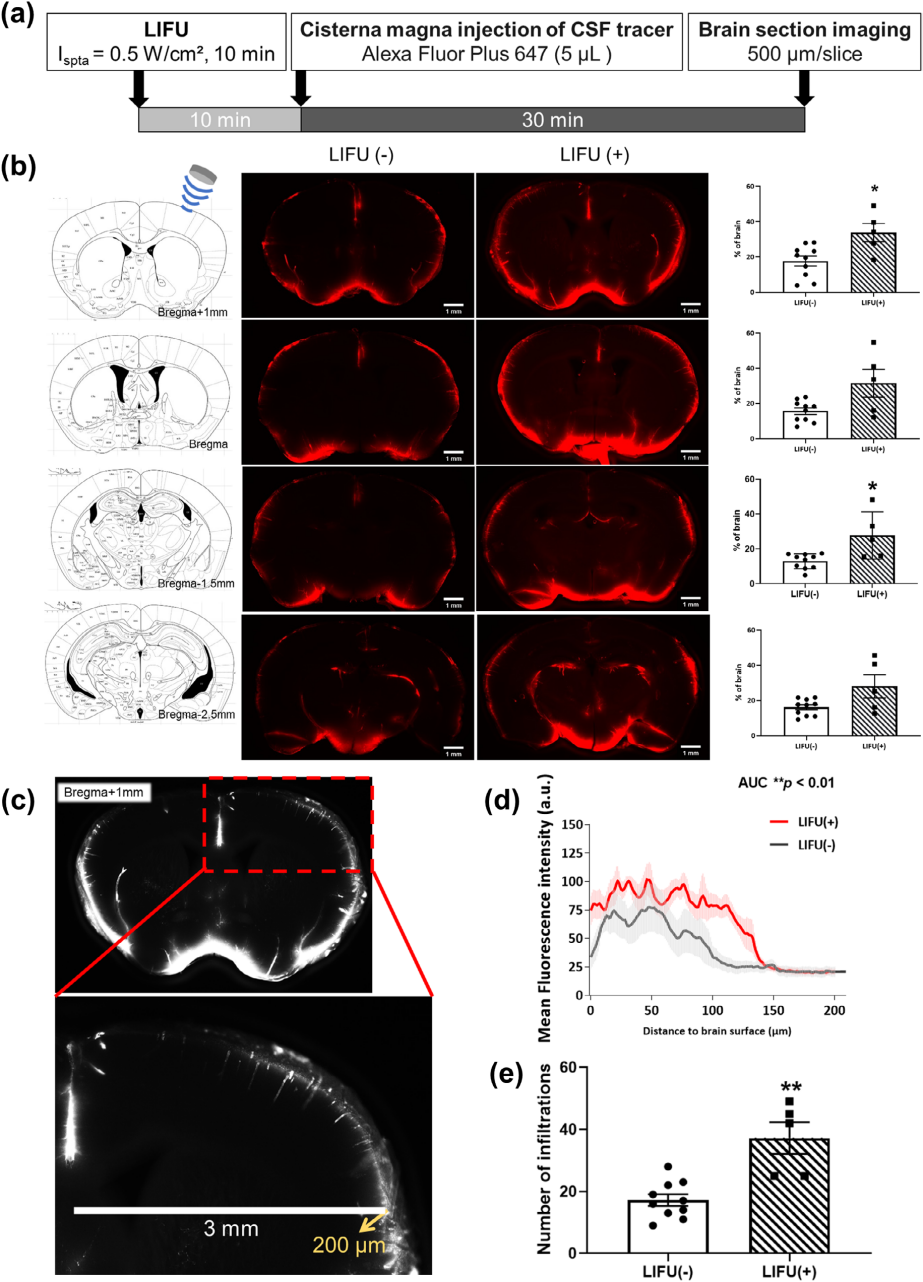
LIFU enhances CSF tracer distribution and penetration in normal mice. Experimental protocol for examining the effect of LIFU on CSF influx (a) Representative coronal brain section imaging 30 min after LIFU and in control (*n* = 10 and 5, respectively) demonstrate an increased percentage of brain area with positive fluorescence of CSF tracer across different sections relative to bregma (scale bar: 1 mm). Significant differences were observed at bregma +1 mm and bregma −1.5 mm levels (b) Tracer penetration depth was calculated at the cortical position 3 mm lateral to the midline, from the pial surface to a depth of 200 μm in the coronal section (bregma +1.0 mm level) (c) The results shown in Figure 1. (d) were analyzed using the area under the curve (AUC) method to assess between‐group differences. Quantification of visible paravascular influx lines at bregma +1.0 mm level (e) The results presented in Figure 1 (b, e) are expressed as mean ± SEM, with error bars representing SEM. An independent *t*‐test was used to analyze differences between groups. Asterisks indicate *p < 0.05 and **p < 0.01.

Thirty minutes after LIFU stimulation, imaging of brain sections showed an increase in the percentage of areas exhibiting enhanced fluorescence intensity. This enhanced fluorescence indicates the distribution of CSF influx into the brain (Figure 1). The percentage of the area with enhanced fluorescence increased by approximately twofold with LIFU compared with the control group in various coronal sections near the stimulation site. The penetrating depth profile showed LIFU increased CSF influx penetration depth from 100 to 150 μm with elevated fluorescence intensity (bregma +1 mm level, calculated at the cortical position 3 mm lateral to the midline). The number of visible paravascular influx lines in the LIFU stimulation area also increased significantly compared with the control group.

### 3.2 LIFU Accelerates the Rate and Increases the Total Intensity of CSF Influx

**FIGURE 2:**
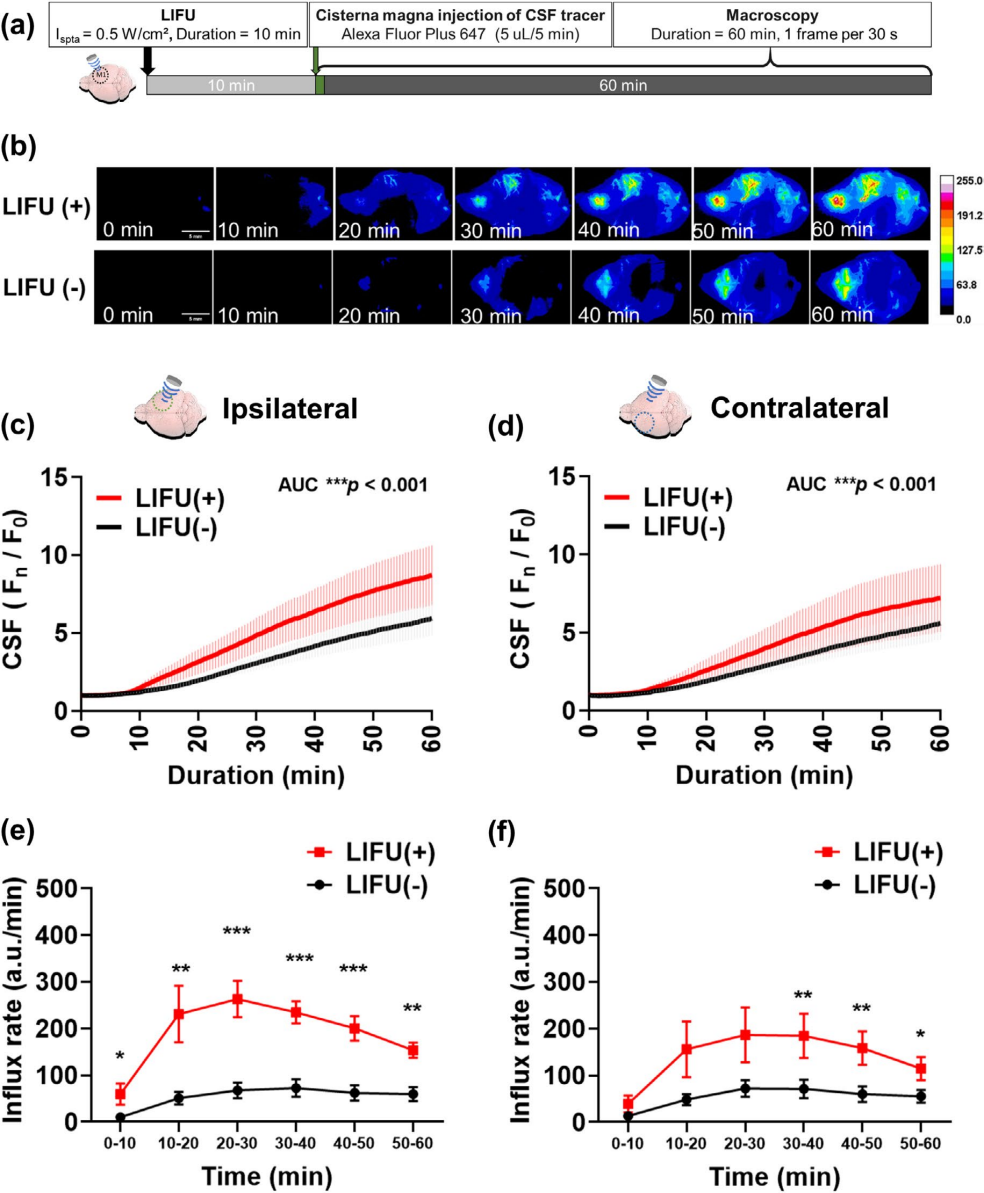
LIFU significantly accelerates the rate and increases the total intensity of CSF tracer influx in normal mice. Experimental protocol for examining the effect of LIFU on CSF influx dynamics (a) Representative in vivo real‐time macroscopic imaging shows enhanced CSF influx after LIFU stimulation, particularly on the stimulation side (scale bar: 5 mm) (b) The fluorescence intensity over time indicates the amount of CSF influx in the ROI ipsilateral (c) and contralateral (d) to LIFU stimulation. The increment of fluorescence intensity over time indicates influx rate in ipsilateral side (e) and contralateral side (f) (*n* = 7 and 14 in LIFU(+) and LIFU(−), respectively). The results shown in Figure 2. (c, d) were analyzed using the area under the curve (AUC) method to assess between‐group differences. Asterisks indicate ***p < 0.001. The results (data presented as mean ± SEM, error bars denoting SEM) shown in Figure 2. (e, f) were analyzed using an independent *t*‐test to assess between‐group differences at different time intervals. Asterisks indicate *p < 0.05, **p < 0.01, and ***p < 0.001. Circular ROIs with a radius of approximately 1.604 mm (area = 8.021 mm^2^) were drawn for analysis.

The fluorescence intensity of the CSF tracer in the cerebrum, representing the amount of CSF influx, showed a significant increase following LIFU stimulation compared with the control group (Figure 2). In the ROI indicating the MCA region on the stimulated side, fluorescence intensity significantly increased in the LIFU group throughout the entire 60‐min observation period. The CSF influx in the contralateral hemisphere showed a more modest but still significant increase compared with the control group.

The increment in fluorescence intensity of the CSF tracer, indicating the CSF influx rate, peaked more rapidly on the LIFU‐stimulated side compared to the control group, with a more than threefold increase at 20–30 min posttracer injection (Figure 2). The CSF influx rate showed a more modest but still significant rise in the contralateral hemisphere, noticeable within 60 min after LIFU stimulation.

### 3.3 CSF Influx Decreases After Photothrombotic Stroke

**FIGURE 3:**
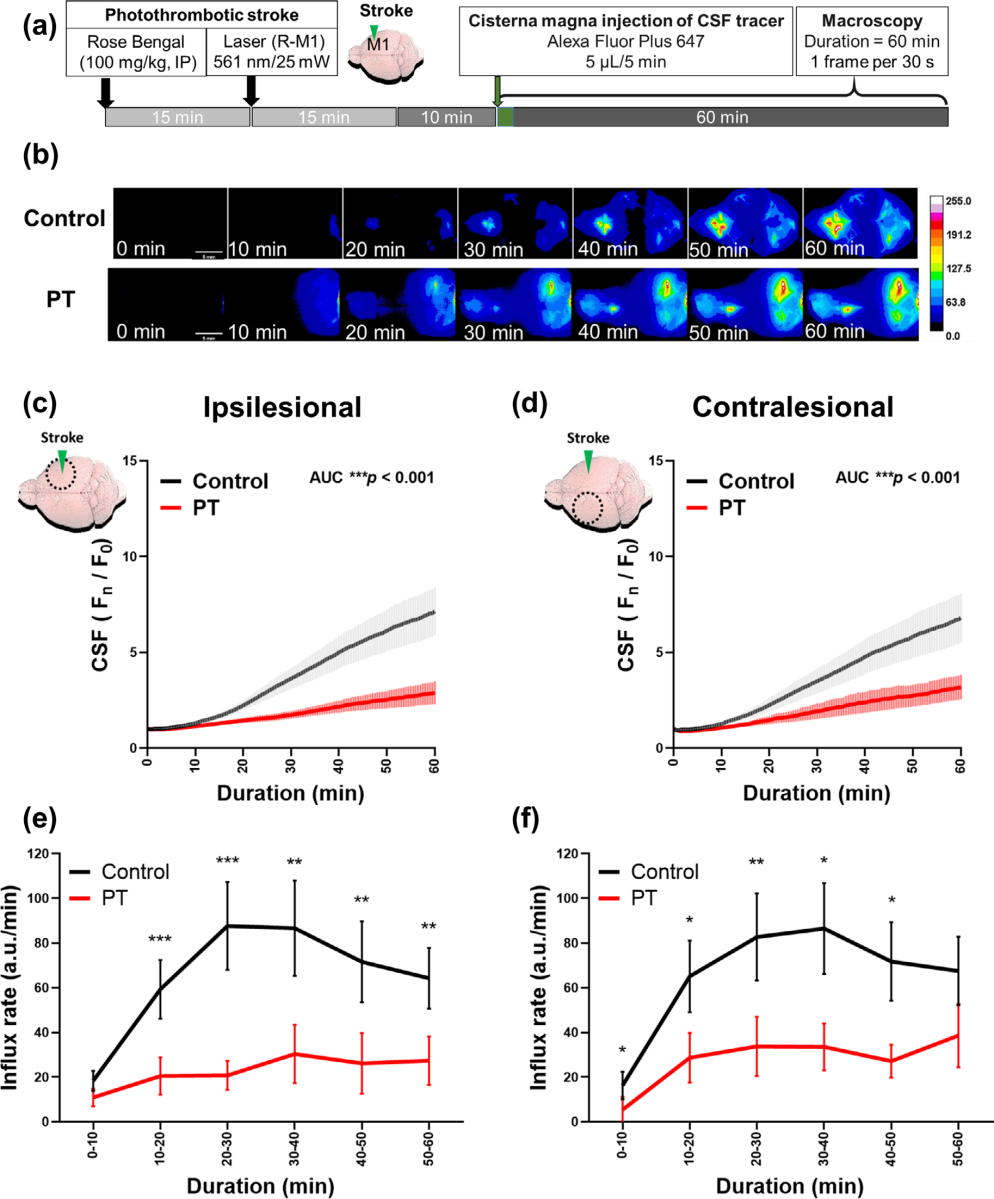
CSF tracer influx was impaired after photothrombotic stroke. Experimental protocol for CSF influx dynamics in the photothrombotic mice (a) Representative in vivo real‐time macroscopic imaging shows impaired bilateral CSF influx following photothrombotic stroke (scale bar: 5 mm) (b) The fluorescence intensity over time indicates the amount of CSF influx in ROI ipsilateral (c) and contralateral (d) to the photothrombotic stroke. The increment of fluorescence intensity over time indicates influx rate ipsilateral (e) and contralateral (f) to the photothrombotic stroke. (*n* = 9 in photothrombotic stroke, *n* = 16 in control group). PT: Photothrombotic. The results shown in Figure 3. (c, d) were analyzed using the AUC method to assess between‐group differences. An asterisk indicates ***p < 0.001. The results (data presented as mean ± SEM, error bars denoting SEM) shown in Figure 3. (e, f) were analyzed using Mann–Whitney test to assess between‐group differences at different time intervals. An asterisk indicates *p < 0.05, **p < 0.01, and ***p < 0.001. Circular ROIs with a radius of approximately 1.604 mm (area = 8.021 mm^2^) were drawn for analysis.

Following a photothrombotic stroke, both the amount and rate of CSF influx decreased on both the ipsilesional side and the contralesional side (Figure 3). The CSF influx intensity in the bilateral ROI was significantly lower after tracer injection in the photothrombotic stroke group compared with the control group. There was minimal CSF influx flow throughout the 60‐min observation period after stroke in both the ipsilesional and the contralesional sides, while the contralateral side showed a trend of late increase in CSF influx rate at 50–60 min.

**FIGURE 4:**
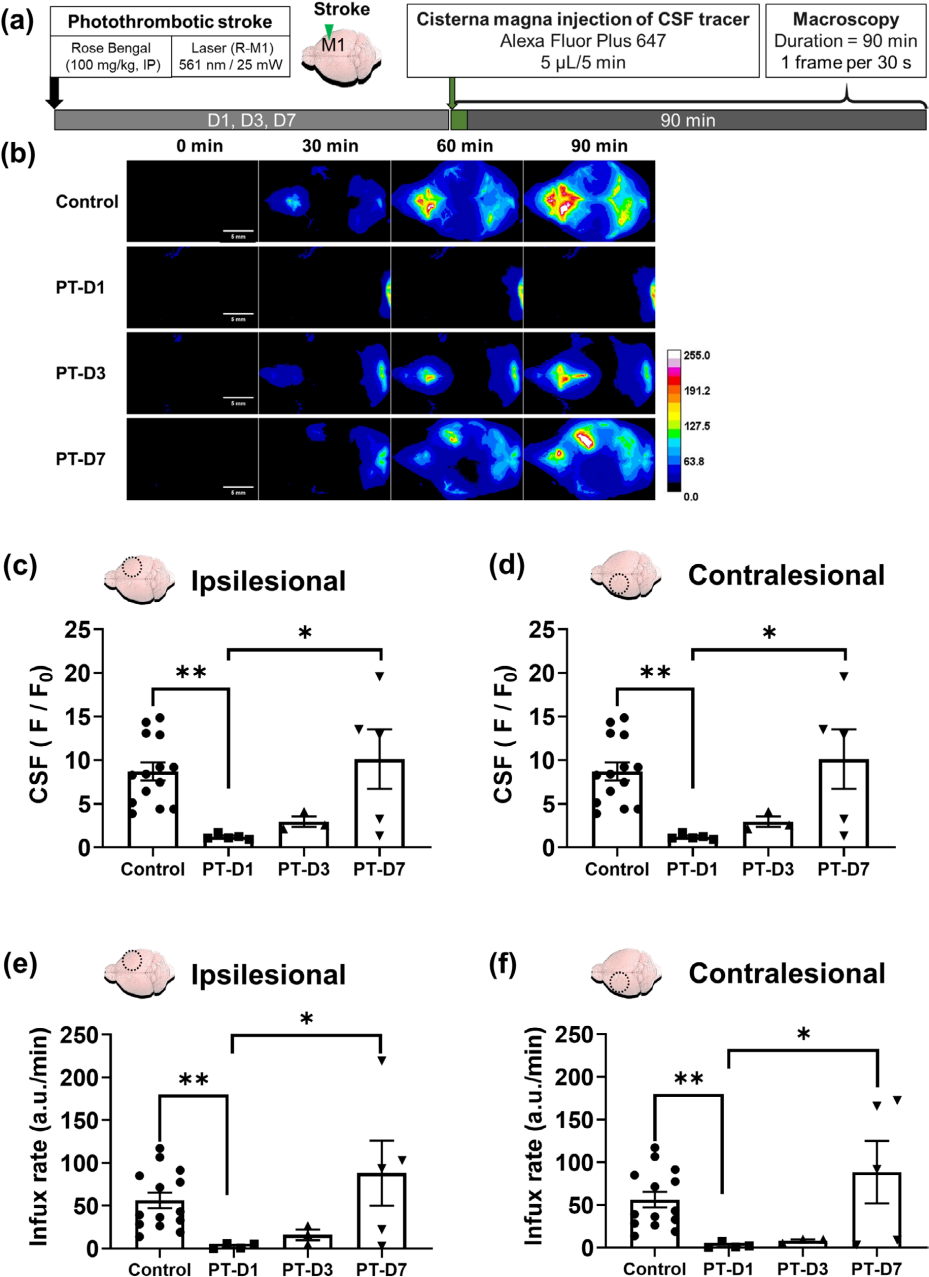
Changes in CSF tracer influx following photothrombotic stroke induction. Experimental protocol for CSF influx dynamics in photothrombotic mice (a) Representative in vivo real‐time macroscopic imaging demonstrates that the fluorescence intensity over time and the influx rate of the CSF tracer were markedly decreased on Days 1 and 3 following photothrombotic stroke (scale bar: 5 mm) (b) Maximum total intensity (30–40 min) (c, d) and peak influx rate (90 min) (e, f) of the CSF tracer showed no difference between the control and PT‐D7 groups. The results (data presented as mean ± SEM, error bars denoting SEM) shown in Figure 4. (c, d, e, f) were analyzed using Kruskal–Wallis test to assess overall differences, followed by post hoc Dunn test to determine between‐group differences. PT‐D1, PT‐D3, and PT‐D7 indicate the Days 1, 3, and 7 following photothrombotic stroke induction, respectively. Asterisks indicate *p < 0.05 and **p < 0.01. Circular ROIs with a radius of approximately 1.604 mm (area = 8.021 mm^2^) were drawn for analysis. Control (*n* = 14), PT‐D1 (*n* = 5), PT‐D3 (*n* = 3), PT‐D7 (*n* = 5).

To investigate the changes in CSF influx over several days following a stroke, a CSF tracer was injected at different time points after stroke induction (1, 3, and 7 days), followed by macroscopy observation. On both the ipsilesional and contralesional sides, the maximal intensity of CSF tracer influx (at saturation of tracer fluorescence intensity) decreased by approximately 8‐fold on the first day poststroke, and gradually returned to baseline levels by Day 7 (Figure 4). Similarly, the peak influx rate, typically occurring 30–40 min after tracer injection, was significantly reduced by 40–50‐folds on Days 1 and 3, and returned to baseline levels by Day 7 (Appendix A Figure).

### 3.4 Contralesional LIFU Restores CSF Influx Impairment Following Photothrombotic Stroke

**FIGURE A2:**
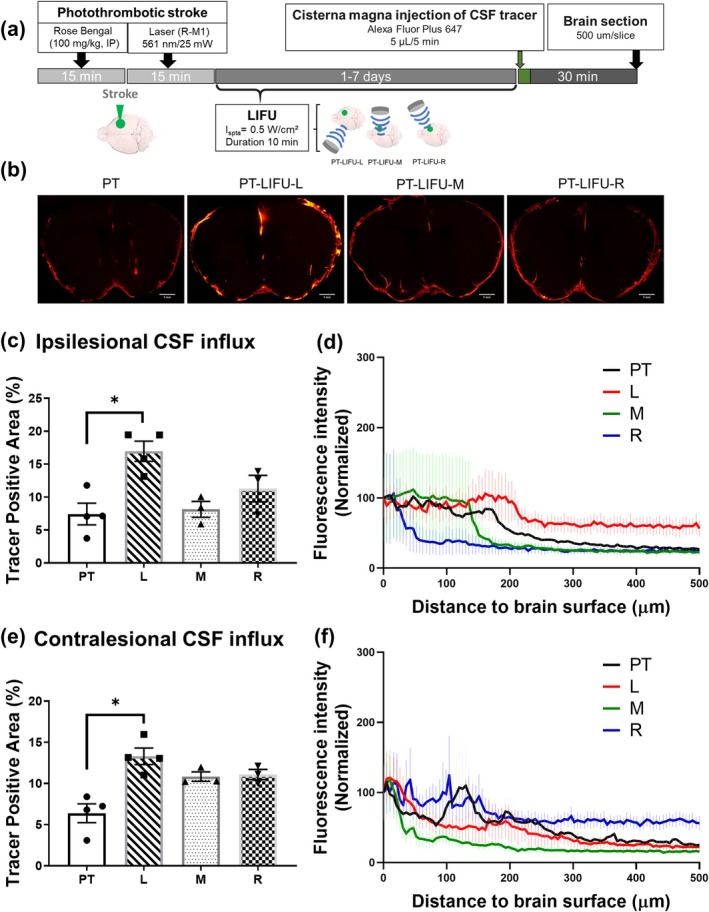
Effect of LIFU stimulation at different brain regions following a photothrombotic stroke. Experimental protocol for assessing the site‐specific effects of LIFU stimulation effect following a photothrombotic stroke (a) The representative brain section (at bregma +0.5 mm) imaging showed CSF tracer influx 7 days poststroke, following daily LIFU stimulation at the contralesional (PT‐LIFU‐L), middle (PT‐LIFU‐M), and ipsilesional (PT‐LIFU‐R) regions (scale bar: 1 mm) (b) The quantification of the percentage of brain area exhibiting positive CSF tracer fluorescence showed enhanced ipsilesional tracer distribution after LIFU stimulation on the contralesional side (c) The contralesional CSF influx distribution was enhanced by LIFU treatment on the contralesional side (e) The penetration depth of the CSF tracer did not show among‐group differences on either side (d, f), as analyzed using the AUC method. The results (data presented as mean ± SEM, error bars denoting SEM) shown in Figure A2 (c, e) were analyzed using the Kruskal–Wallis test to assess overall differences, followed by post hoc Dunn test. An asterisk indicates *p < 0.05. PT‐control (*n* = 5), PT‐LIFU‐L (*n* = 4), PT‐LIFU‐M (*n* = 3), PT‐LIFU‐R (*n* = 3).

To investigate the site‐specific therapeutic effects of LIFU, the treatment was applied to different regions of the brain (ipsilesional M1, contralesional M1, and central cerebrum) for seven consecutive days after inducing photothrombotic stroke. Brain section imaging conducted 7 days poststroke revealed a global decrease in CSF influx distribution in the bilateral hemispheres (Appendix A Figure A2). The CSF influx impairment on both the ipsilesional and contralesional sides was reversed only by contralesional LIFU treatment, but not by treatments applied to other sites.

**FIGURE 5:**
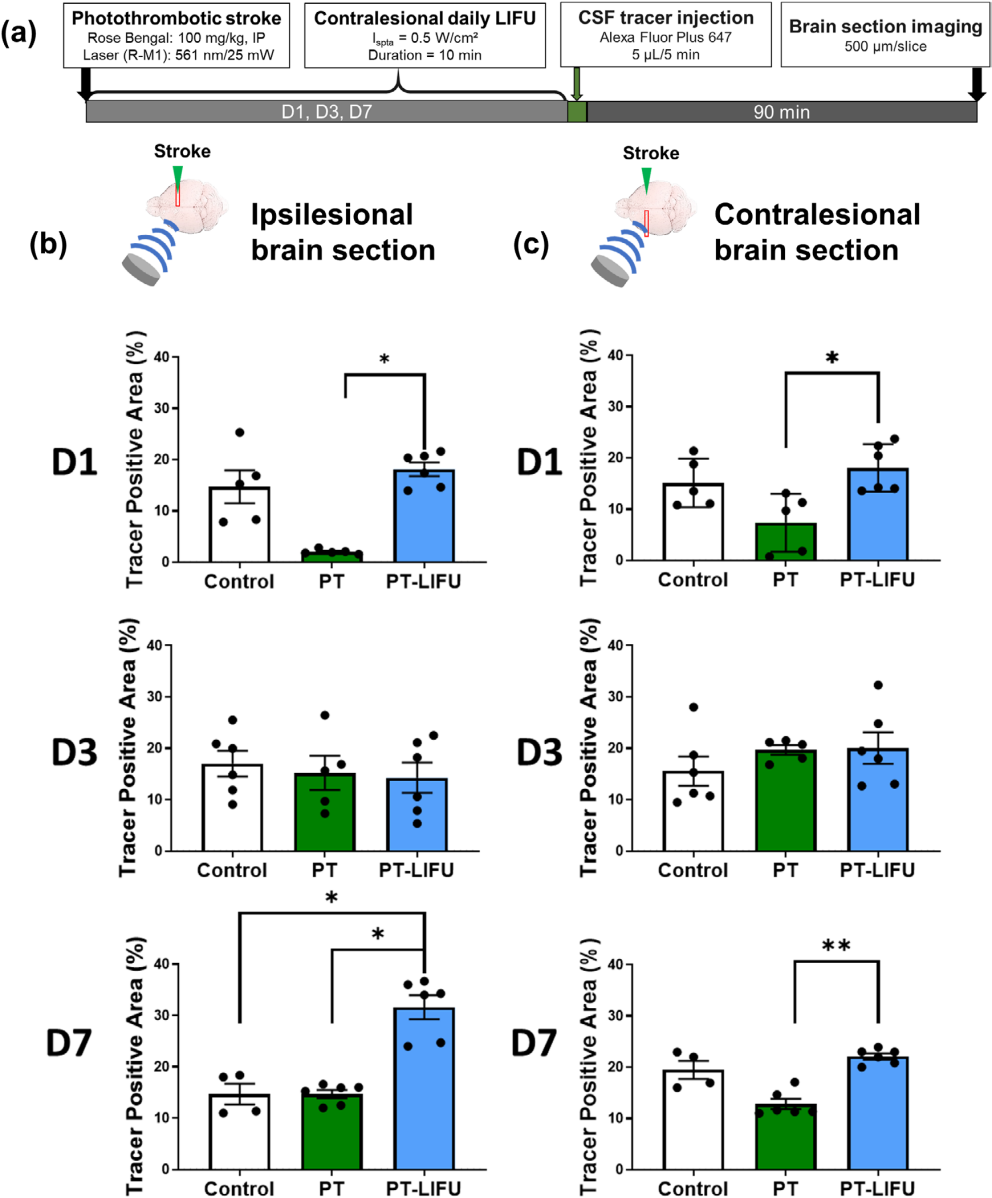
Contralesional LIFU restore impaired CSF influx following photothrombotic stroke. Experimental protocol for daily LIFU treatments following photothrombotic stroke (a) The percentage of brain area with positive fluorescence of CSF tracer on the stroke side (b) and on the contralesional side (c) LIFU was applied to the contralesional side. The results (data presented as mean ± SEM, error bars denoting SEM) shown in Figure 5. (b, c) were analyzed using Kruskal–Wallis test to assess overall differences, followed by post hoc Dunn test to determine between‐group differences. Asterisks indicate *p < 0.05 and **p < 0.01. Day 1: Control (*n* = 5), PT (*n* = 5), PT‐LIFU (*n* = 6); Day 3: Control (*n* = 6), PT (*n* = 5), PT‐LIFU (*n* = 6); Day 7: Control (*n* = 4), PT (*n* = 5), PT‐LIFU (*n* = 6).

Based on the finding that only contralesional LIFU restored the ipsilesional CSF influx, LIFU was applied to the contralesional side for one, three, or seven consecutive days following stroke induction. Brain section imaging showed that on Day 1, the CSF influx decreased on the ipsilesional side and was restored by contralesional LIFU (Figure 5). On Day 3 there was no significant difference among the groups, but consecutive LIFU stimulation resulted in a further increase of CSF influx on the ipsilesional side on Day 7.

The contralesional CSF influx decreased to a lesser extent and was also increased by LIFU on the same side. Overall, the reduced CSF influx after stroke was restored by LIFU at Day 7 (Figure 5).

## 4 Discussion

Our study revealed a significant enhancement in CSF influx following localized LIFU stimulation at the M1 region. This effect manifested rapidly, with an increased CSF influx rate observed within 10–20 min, reaching peak flow at 30–40 min post LIFU stimulation. Following the induction of photothrombotic stroke, a substantial reduction in CSF influx was evident at both the ipsilesional and contralesional MCA regions. This impairment was most pronounced at Days 1 and 3 after stroke, gradually returning to baseline levels by Day 7. Notably, daily contralesional LIFU stimulation over a 7‐day period effectively restored the impaired CSF influx on both sides. These dynamic insights into CSF influx alterations induced by LIFU, especially in the context of regional stroke, highlight the potential implications for stroke‐related pathophysiology. This study provides a foundation for further exploration of LIFU as a modality for targeted therapeutic interventions in stroke recovery.

This study represents a comprehensive exploration of the alterations in CSF influx following stroke and their potential reversal through LIFU intervention. Previous research has already hinted at the potential of transcranial LIFU without microbubbles to modulate the glymphatic system in nondisease murine models, as evidenced by short‐term enhancements in CSF influx observed by brain section imaging [14]. Additionally, LIFU has been employed to enhance intracortically injected CSF tracer transport and improve its clearance [22], albeit with a limited duration of observation. A recent study in mice observed increased perivascular CSF influx following LIFU, targeting the center of the brain (at the 3rd and 4th ventricles) as demonstrated by two‐photon imaging [13]. Our study extends these findings by showing the dynamic change of CSF influx at bilateral hemispheres after unilateral LIFU stimulation at the M1 cortex. Unilateral LIFU accelerates the rate and increases the total CSF influx intensity bilaterally. It may have therapeutic implications, as targeting the nonaffected side in stroke or brain injury could carry less risk while still promoting recovery.

Although the ultrasound parameters in our study differ somewhat from those used in previous research that reported increased CSF tracer movement [13, 14, 22], we applied a similar intensity and duty cycle, resulting in the same I_spta_ of 0.5 W/cm^2^. While the previous study used longer stimulation durations (30 min to 1 h), we limited ours to 10 min to better mimic clinical conditions and enhance its translational potential. Additionally, the earlier study used a constant 100 ms pulse duration per second, resulting in a 10% duty cycle. In contrast, we applied a pulse train duration of 300 ms (2 ms pulse duration, 50% pulse train duty cycle) with an interstimulus interval of 2 s, achieving an overall duty cycle of 7.5%. Our approach was based on previous LIFU neuromodulation studies [21], utilizing parameters characterized by intermittent pulses to emphasize the mechanical effects of ultrasound. Overall, the intermittent pulses, applied with a similar intensity shown to cause no damage in previous studies, along with a short stimulation duration, offer greater translational potential.

Our results showed that the impairment in CSF influx was most prominent at Days 1 and 3 after stroke and gradually returned to normal afterwards. The impairment of the glymphatic system following a stroke has been noticed in several previous studies. In a mouse model of acute embolic stroke, both MRI and histological examinations have shown a compromised CSF influx into the ipsilateral cortex as early as 3 h after occlusion of the middle cerebral artery. Moreover, recanalization leads to the restoration of CSF inflow [4]. In other mouse models of photothrombotic stroke and MCAO, both CSF influx and efflux in both the ipsilateral and contralateral hemispheres were reduced 24 h after the stroke. This reduction may contribute to an increase in intracranial pressure following the stroke [23, 24]. Similarly, in mouse models and a nonhuman primate model of subarachnoid hemorrhage, impaired CSF inflow has been observed along periarterial influx pathways, with noticeable effects emerging 24 h after the injury occurs [25, 26]. We further demonstrated that CSF influx reaches its nadir on Days 1 and 3 after stroke. Therefore, assessing whether daily LIFU treatment can reverse CSF impairment in the days following stroke is crucial, as it may have significant therapeutic implications.

Our study provides evidence that LIFU, particularly when applied at the contralesional hemisphere, may help restore impaired CSF influx after an ischemic stroke. Additionally, consecutive LIFU for 7 days further improves the CSF influx. The effects were most evident on Day 1, but by Day 3, there was no significant difference in CSF influx between the stroke and control groups, possibly due to the rapid recovery of the mice. However, by Day 7, daily LIFU treatments further enhanced CSF influx. By facilitating CSF circulation, LIFU has the potential to mitigate brain edema and secondary ischemic injuries. A recent study in mice with MCAO showed that brain edema peaked on Day 2 after MCAO, with the time course of edema progression closely aligning with glymphatic system dysfunction [24]—findings consistent with our results. The study also demonstrated that pharmacological enhancement of glymphatic function significantly alleviated brain edema after MCAO. However, the enhancement achieved through intraperitoneal administration of adrenergic inhibitors in their study may result in systemic effects, limiting its clinical applicability. In contrast, LIFU offers a more localized therapeutic approach, with the potential to enhance CSF influx while minimizing systemic adverse effects, thereby presenting a potentially superior strategy for managing brain edema. Importantly, our findings indicate that consecutive LIFU treatment over a 7‐day period significantly improves CSF influx, implying its potential utility in subacute stroke therapies.

Our study has several clinical implications. First, impaired glymphatic circulation after stroke has been observed in humans. In an MRI study involving human stroke patients, the analysis of DTI‐ALPS, which represents perivascular CSF flow, showed a significant decrease on the side of the infarct compared with the contralateral side [6]. Impaired glymphatic circulation is linked to an increase in intracranial pressure, consequently leading to reduced microcirculation. This, in turn, worsens ischemic injury [27]. Modulating the glymphatic system presents a therapeutic opportunity, such as enhancing circulation to support recovery. Second, we demonstrated that regional LIFU accelerates bilateral CSF influx. Targeting the nonaffected side in stroke treatment may pose less risk while still promoting recovery, offering a safer approach to improving outcomes. Finally, consecutive LIFU for 7 days further enhanced CSF influx, suggesting its potential application in the treatment of subacute stroke.

### 4.1 Study Limitations

This study has certain limitations that should be acknowledged. Firstly, our investigation primarily focused on CSF influx, lacking comprehensive data on efflux or clearance mechanisms. Additionally, our observation period for the CSF tracer was confined to 60–90 min, when the fluorescence intensity reaches saturation, providing a snapshot of the short‐term dynamics. Long‐term and continuous data are crucial for a more thorough understanding of CSF influx dynamics, particularly after stroke. Furthermore, while we successfully explored the phenomenon of LIFU enhancing CSF influx, we did not delve into the underlying mechanisms. Although the exact mechanism remains unclear, a study suggested that LIFU reduces brain edema formation in rats, potentially by influencing the localization of aquaporin‐4 (AQP4) on cell membranes [28]. One study showed that the TRPV4‐calmodulin‐AQP4 axis plays a role in very low‐intensity ultrasound‐mediated glymphatic enhancement [29]. Future studies should aim to correlate the observed effects with outcomes such as brain edema, infarction volume, and animal behavior scores. Histological assessments could provide valuable insights into the cellular changes associated with LIFU‐mediated glymphatic restoration after stroke. Addressing these aspects will contribute to a more comprehensive understanding of the potential implications of LIFU in the context of stroke treatment.

## 5 Conclusions

Our study shows the rapid enhancement of CSF influx post LIFU stimulation at M1. After inducing stroke, CSF influx significantly reduced in both the ipsilesional and contralesional MCA regions, with the greatest reduction on Days 1 and 3, and returning to baseline by Day 7. Daily contralesional LIFU over a 7‐day period effectively restored impaired CSF influx on both hemispheres. These findings collectively underscore the potential of ultrasound‐based approaches for modulating CSF dynamics, particularly in the context of stroke treatment.

## Author Contributions

M.‐Y.H. conceived the idea, analyzed the data, and was a major contributor to writing the manuscript. Y.‐L.L. conducted the experiments, analyzed the data, and drafted the manuscript. M.‐T.L. and W.‐H.L. analyzed the data and contributed to manuscript drafting. W.‐S.C. and C.‐H.W. supervised the study and revised the manuscript. All authors read and approved the final version.

## Conflicts of Interest

The authors declare no conflicts of interest.

## Figures and Tables

**FIGURE A1:**
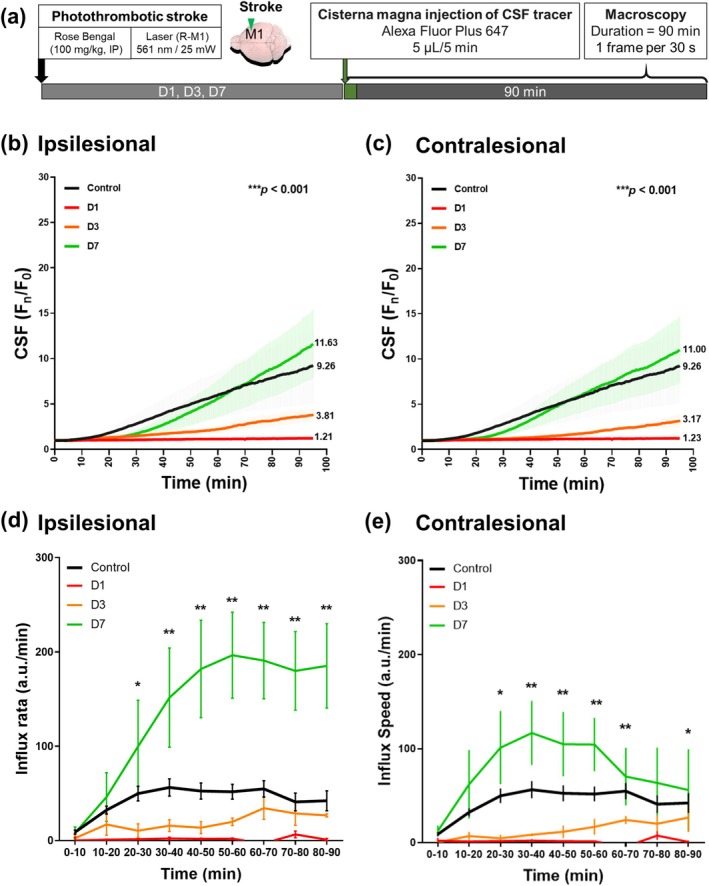
CSF influx on Days 1, 3, and 7 following photothrombotic stroke. Experimental protocol for CSF influx dynamics in photothrombotic mice (a) The fluorescence intensity over time (b, c) and the influx rate (d, e) of the CSF tracer were markedly decreased on Days 1 and 3 after photothrombotic stroke. The results shown in Figure A1 (b, c) were analyzed using the area under the curve (AUC) method to assess between‐group differences. The results (data presented as mean ± SEM, error bars denoting SEM) shown in Figure A1 (d, e) were analyzed using Kruskal–Wallis test to assess overall differences, followed by post hoc Dunn test to determine between‐group differences. Asterisks indicate *p < 0.05 and **p < 0.01. Circular ROIs with a radius of approximately 1.604 mm (area = 8.021 mm^2^) were drawn for analysis. Control (*n* = 14), D1 (*n* = 5), D3 (*n* = 3), D7 (*n* = 5).

## Data Availability

The data that support the findings of this study are available from the corresponding author upon reasonable request.
